# Phospholipid and Lipid Derivatives as Potential Neuroprotective Compounds

**DOI:** 10.3390/molecules23092257

**Published:** 2018-09-05

**Authors:** Seyed Khosrow Tayebati

**Affiliations:** School of Pharmacy, University of Camerino, 62032 Camerino, Italy; khosrow.tayebati@unicam.it; Tel.: +39-073-740-3305

**Keywords:** brain, phospholipids, choline derivative lipids, neuroprotection, neurotoxicity

## Abstract

The worldwide demographical trend is changing towards a more elderly population. In particular, this phenomenon is increasing the number of neurodegenerative disease cases (e.g., Alzheimer’s disease) in advanced countries. Therefore, there is a fertile field for neuroprotective approaches to address this problem. A useful strategy to protect the membrane integrity of cells and reduce inflammatory processes. In this context, the neurons represent particularly vulnerable cells. Thus, a protection strategy should include their membrane preservation and improved anti-inflammatory processes. The contribution of phospholipid derivatives to this issue is crucial and many articles evidence their role in both health and disease. On the other hand, some lipids containing choline actively participate to increase the choline levels in the nervous system. It is acknowledged that the cholinergic system plays a pivotal role both in the central and in the peripheral nervous system. Neurons cannot synthesize choline, which is provided by the diet. The reuptake of ACh and its hydrolysis represent the principal source of choline. Therefore, to cover choline needs, choline-containing lipids may be used. There are different works which demonstrate their neuroprotective features This review article analyzes phospholipid and lipid derivatives that through different mechanisms are involved in these protective processes, although, sometimes the same molecules may behave as neurotoxic elements, therefore, their protective machinery should be detailed better.

## 1. Introduction

Because of the high morbidity rate of neurodegenerative diseases, the neuroprotection strategy looks like a promising method to address this problem. The elevated numbers of neurological damage cases among the world populations is in part due to the high numbers of elderly people, both in Western countries and in other countries such as Japan, China, etc. 

Alterations in lipid metabolism including activation of phospholipases and release of arachidonic acid (ArAc) are key events that contribute to neuronal death in cerebral ischemia [1,2,3,4]. It is well known that neuronal membranes contain phospholipid pools that are the source for the synthesis of specific lipid couriers on the basis of neuronal stimulation or injury [5]. Actually, these molecules, in turn, participate in signaling cascades that may promote either neuronal injury or neuroprotective effects [5]. In addition, the phospholipids participate in different ways to improve cell membrane state, preventing neuronal death both in “in vitro” and in “in vivo” models of ischemia [6]. 

Other lipids that are actively involved in neuron survival belong to the lysophospholipids family. Many of these molecules were used both in “in vitro” and in “in vivo”, with significant effects regarding neurons after an ischemic event [6]. On the other hand, the bioactive lipid involvement in the regulation of synaptic function/dysfunction is also well established [7,8]. The anti-apoptotic action of phospholipase A2 is also documented. Actually, these molecules reduce neuronal cell death, apoptosis and promote cell survival in a murine model of stroke [9]. 

In this review, the role of different lipid compounds in the cellular survival or potential cellular damage is evaluated. Among the studied lipid molecules, there are sphingolipids, bioactive lipids, cholesterol, docosahexaenoic acid (DHA), membrane phospholipids and choline-containing lipids. Their protective and/or neuroprotective effects or their possible negative roles in neuronal cells are discussed.

## 2. Sphingosine, Its Receptors, and Their Functions

Sphingosine-1-phosphate (S1P) is a bioactive signaling molecule regulating cell proliferation and survival as well as differentiation and motility [10,11]. It derives from degradation of sphingolipids via the cleavage of ceramide into fatty acid and sphingosine [12]. S1P can be dephosphorylated by sphingosine phosphatases back to sphingosine and thereby recycled for ceramide formation [13]. Its protective role against radiation, chemotherapy and endothelial apoptosis has been investigated in several studies [14,15,16]. It is one of a multitude of sphingolipids and glycosphingolipids that are readily synthesized and/or inter-converted in a spatial and temporal manner in response to environmental changes and stimuli [17,18]. 

S1P is an intriguing lipid metabolite. Actually, it also works as a ligand of S1PR1 to S1PR5, a group of five G protein-coupled cell surface receptors. Interestingly, these receptors are expressed on different cells of the immune, cardiovascular, respiratory, hepatic, reproductive, or neurologic systems (Table 1 and Table 2) [19,20,21,22,23,24,25,26,27,28]. On the other hand, S1P displays different behaviors in various patho-physiological conditions including autoimmune, cardiovascular, cancer, deafness, osteogenesis, reproduction and, neurodegenerative diseases [28].

Another sphingosine family that actively participate in cell damage and inflammatory events are the sphingosine-1 kinases (SPhK 1). These molecules regulate the expression of cytokines involved in pro-inflammatory activities [29]. In a more recent study the role of SPhK 1 and S1P in human dopaminergic cells treated with MPP+ was evaluated [30]. The results of this work are essentially in contrast with some studies in which pro-inflammatory effects of these compounds on glial cells were described [29].

Membrane lipid metabolic anomalies frequently occur in the elderly and may be an important cause of neurodegenerative diseases [31]. Different age-dependent neurodegenerative dysfunctions, such as Alzheimer’s disease (AD), Parkinson disease (PD), or cerebral ischemia may be also influenced directly by a disordered sphingolipid metabolism. This process could occur by means of different effects of S1P on a number of neuron activities (e.g., generation, endurance, neurite retraction/extension, neurotransmitter release [32,33] and its complex functions related to oxidative stress-linked events [34,35].

Sphingosine kinases (SphKs) are subject to accurate control by post-translational modifications [36], differential subcellular translocation [36,37], coupling partners [38,39], and have partially distinct objectives [37,40]. Sphingolipid signaling may affect amyloid beta (Aβ) amount [41,42] and siRNA/inhibition of SphK1/SphK2 decreases β-split of Amyloid Precursor Protein (APP) leading to lower the production of Aβ and sAPPβ [42]. SphKs and sphingolipids could also manage Aβ toxicity [43,44] and the inflammatory process (due to it) [45] as well as the consequent cerebral parenchyma damage [46]. Alternatively, Aβ blocks S1P generation [44] and regulates its receptors [45]. Actually, an association between sphingolipid irregularities and cognitive impairment was noted in AD [47] and the protective effect of SphKs in Aβ toxicity has been described [44]. The effects of SphK inhibitors on APP secretion was evaluated [48]. Actually, SphK and S1P may be considered as pro-secretory compounds in APP secretion.

The neuroprotective properties of S1P receptor signaling in the AD, cerebral lesion, ischemia and other neurological dysfunctions are established [42,46]. By contrast, there is limited data about its involvement in glucose deprivation/glucose reload (GD/GR) stress. S1P, the product of Sphk1/2 activity, is also a bioactive lipid intercessor that promotes cell outliving, amplification, migration, and angiogenesis.

Irregularity in gene expression for Sphk1/Sphk2 and sphingosine-1-phosphate receptor 1 (S1P1) in the animal model of cerebral ischemia was observed [11]. In the same study, the effects of an S1P analog, pFTY720, on the neuronal injury reduction was checked in cerebral ischemia [11]. As previously quoted, anti-apoptotic effects of S1P in response to oxidative stress induced by H_2_O_2_ were detected [49]. It was noted that the S1P’s anti-apoptotic effect is mediated through PI3K/Akt signaling and is modulated by means of receptors S1P1 and S1P3 [49]. On the other hand, the S1P analog, pFTY720, and the specific agonist of S1P1, SEW2871, increase cell viability under GD/GR stress [50]. 

Actually, some studies have identified the neuroprotective role of S1P through mitochondria in an oxygen-glucose deprivation model [32]. This study mentioned that S1P treatment significantly decreased both necrosis and apoptosis in the in vitro model of ischemia [32]. Its protective mechanisms concern stabilization of the mitochondrial membrane potential, reduced calcium loading and decreased sensitivity to mitochondrial permeability transition pore opening. The mitochondrial outer membrane permeabilization seems important for the S1P protective effect. Actually, it is dependent on B-cell lymphoma-2 (Bcl-2) proteins, which control a critical step in the commitment to apoptosis.

Sphingosine-1-phosphate receptors (S1PRs) are a member of the G protein-coupled family of receptors. They are known drug targets for some neurological disorders like multiple sclerosis (MS) [51,52,53,54]. Actually, S1PRs represent five members (S1P1-S1P5). S1P is considered the endogeneous ligand for these receptors. In fact, it was found in different concentrations in the body fluids and tissues [53]. An abnormal concentration of S1P in some inflammatory sites may be connected to pathological processes [53].

### 2.1. S1PRs Biological Significance

The biological importance of S1PRs in the CNS is widely studied [55]. These receptors are expressed both in neuronal and glial cells (Table 2). Their level of expression may be variable because of temporal and spatial factors as well as stimuli surrounding the cell. It is established well enough that S1PRs effects, in the CNS, primarily regard morphological changes related to growth cone formation and neurite extension and retraction [55]. Sometimes, a different expression of these receptors can have opposing significance. A clear example of this process may be represented by the overexpression and application of antisense probes to down-regulate S1PRs where S1P1 promotes neurite extension, while S1P2 and S1P5 signaling inhibits this procedure [56,57]. A functional role of some S1PRs was also demonstrated. Actually, neurons lacking S1P2 led to elicit hyper-excitability, suggesting an important role of S1P2 in neuronal activity [58]. S1PRs, at least partially, regulate also the synaptic activity.

This kind of regulation, besides of neuronal viability and neurogenesis, may be an important factor to support the utility of these receptors as drug targets in psychiatric disorders and memory impairments, as well as their benefits in multiple sclerosis.

The contribution of S1PRs in the glial cells is quite crucial. Among these cells, oligodendrocytes represent more interested in S1PRs’ function. Actually, the S1PR expression varies in the oligodendrocytes depending on their maturation and myelinating state.

### 2.2. S1P, Neurodegeneration, and Brain

S1P is considered a bioactive lipid that may play a double role in the nervous system [59]. Actually, it is essential for brain development, but its detrimental effects on some particular neuron cells were also revealed [59]. The regulation of its concentration in the cerebral areas probably suggests a particular function of S1P. This kind of regulation, often, is governed by specific kinases and phosphatases or its degrading enzyme, S1P-lyase. Intriguingly, the latter compound deficiency also induces hyperphosphorylation of tau [60], and altered metabolism of the amyloid precursor protein (APP) leading to increased generation of amyloid-β (Aβ) [61]. On the other hand, the importance of S1P levels to govern Aβ accumulation was also demonstrated by S1P-lyase manipulation [61]. Actually, these data evidenced the S1P-lyase’s relevance in the cellular metabolism of APP. Furthermore, this molecule may modulate the Aβ generation and therefore it could be considered as a target to fight the Aβ accumulation. In a quite recent (postmortem) study on AD patients, the authors have provided evidence of a deregulated S1P balance in capillary cerebral amyloid angiopathy (capCAA) [62]. Therefore, further studies are needed to elucidate the role of S1P in capCAA and, AD.

### 2.3. S1P Involvement in Health and Disease

Recently, the role of S1P and other bioactive sphingolipids (e.g., ceramide) in cellular homeostasis was extensively studied [63]. Actually, some enzymes (e.g., ceramidases) represent a series of key enzymes that manage the concentration of bioactive lipids as S1P. Furthermore, it is quite clear that these enzymes are involved in the government of different biological processes (e.g., cell growth and differentiation, autophagy and apoptosis). Therefore, it may be clearly explained that the cause of some important disorders such as neurodegenerative diseases may reside in a dysregulation of these enzymes due probably to their gene mutation. Consequently, as was hoped for the role of S1P in capCAA and, AD, more studies that are specific should be arranged to identify novel potential mechanisms for developing efficacious strategies to challenge these terrible diseases. 

The peripheral role of S1P was also widely studied. Actually, its involvement in inflammatory bowel disease (IBD) seems clear [64]. The S1P receptors play an important role in IBD if they are properly modulated. Being small molecules these molecules may offer many advantages in the care of IBD [65]. An important advantage of these “drugs” could be their oral administration that may avoid the anti-drug antibodies formation that causes injectable biologic therapies to fail when used in IBD treatment [65].

## 3. Bioactive Lipids (Eicosanoids and Endocannabinoids)

The bioactive lipids, based on their biochemical roles, may be divided into four principal classes: classical eicosanoids, specialized pro-resolving mediators (SPMs), lysoglycerophospholipids/sphingolipids and endocannabinoids (eCBs). They originate from ω-6 or ω-3 essential polyunsaturated fatty acid (PUFA) precursors. 

The main role of these bioactive lipids regards their involvement in the inflammatory status modulation. Actually, classical eicosanoids represent highly pro-inflammatory compounds. This kind of pro-inflammatory mechanism should end as soon as the injurious stimuli are terminated.

Intriguingly, however, some bioactive lipids stop the pro-inflammatory action of other lipid molecules. In fact, these compounds cease inflammation and lead to the reinstatement of full tissue homeostasis by activating the signs of resolution: removal, relief, restoration, regeneration, and remission [66,67]. It is quite clear that the bioactive lipid’s action may play an important role in chronic inflammation leading to many pathologies, such as cancer, autoimmune, metabolic, cardiovascular, and neurodegenerative diseases [68,69]. Therefore, it looks like these molecules actively participate in the inflammation intensity management, either operating as fire-starters or as fire-fighters or even as managers of the fire station [70]! 

Bioactive lipids belong to different families in relation to their linkage with other compounds such as ethanolamine, choline, inositol, serine or and fatty acids (e.g., phosphoinositides, lysoglycerophospholipids, and ceramides).

Among bioactive lipids, there are also lysophospholipids, sphingolipids, and endocannabinoids (eCB). These molecules are often involved in the inflammatory processes. Therefore, it is necessary to discover if, during an inflammatory event, they coexist, and thus, in that case, if and how they interact with the inflammatory substrate alone or in combination between them. Undoubtedly, both eicosanoids and SPMs participate in all of the inflammation stages, operating with different paths. Actually, eCB levels quickly increase after a harmful stimulus. Thus, initially this may be considered a protective mechanism but, unfortunately, extension of an inflammatory status leads to eCB system dysregulation that may cause some detrimental effects! Finally, different evidence not only confirms the coexistence of different bioactive lipids, but also suggest that each inflammatory condition needs the concerted involvement of such lipid mediators, which probably also interact molecularly and participate in physiopathological cross-talk events [71,72,73,74].

## 4. Cholesterol and DHA

An opposite effect of LDL cholesterol and DHA in an AD-like pathology animal model is clearly demonstrated [75]. It looks like a DHA-enriched diet can inhibit AD-like disease in an animal model [75]. This beneficial action is probably dependent on the Aβ concentration in the cerebrovascular network. Actually, in this model, the relative cerebral volume was modestly increased. Therefore, some dietary lipids may modify cerebral hemodynamics before affecting the brain Aβ concentration and its detection. Probably, these changes could influence the course of the disease [66]. The mechanisms of this “protective function” of DHA should be examined in depth since this important effect does not apply to every kind of lipid. Actually, in line with the latter statement, the DHA protective effect at the synaptic level was also reviewed and different mechanisms including oxidative stress reduction and inflammatory processes inhibition were proposed [76]. Furthermore, the nutritional contribution of DHA was evaluated [68]. A number of such findings were reviewed [77]. These data have clearly demonstrated that the presence of DHA and its involvement in synaptic membrane and photoreceptors contribute to an improvement of different functions such as vision, neuroprotection, successful aging, and cognitive function [77]. Actually, an esterified DHA form was abundantly detected in the CNS [78]. On the other hand, the concomitant protective effect of enriched DHA diet and the inhibition of DHA-containing phospholipids hydrolysis was evaluated. The results of this study suggest that both DHA and the inhibition of DHA-containing phospholipid hydrolysis lead to potent protection against neurodegeneration after hypoxia/hypoglycemia [79]. These findings confirm that the beneficial neuroprotective effects of DHA-enriched diets might depend on the complex mechanisms, which include anti-oxidative and anti-inflammatory activities of DHA-containing phospholipids, free DHA, and physiologically active DHA derivatives.

## 5. Other Membrane Phospholipids 

The membrane phospholipids (phosphatidylcholine (Ptcho), phosphatidylethanolamine (Ptetn), and phosphatidylserine (Ptser)) are transformed by phospholipase A_2_ (PLA_2_). Actually, PLA_2_ hydrolyzes ArAc at the sn2 position of different membrane phospholipids [80,81].

Cytidine 5′-diphosphocholine (CDP-choline or citicoline) is an endogenous intermediate in the biosynthesis of neuronal membrane phospholipids [42,82,83,84] and works as a choline donor. A different hypothesis was proposed regarding its ability to alter phospholipid metabolism in the treatment of cerebral ischemia [84,85]. It also was demonstrated that CDP-choline treatment attenuated ArAc release after ischemia/1-day reperfusion [83,84]. Another hypothesis may intuitively involve the Ptcho synthesis. The latter hypothesis was evaluated and it was postulated that the Ptcho may be increased by two different pathways: a) transfer of phosphocholine to 1,2-diacylglycerol to form Ptcho and b) choline liberated from CDP-choline that can be converted to S-adenosyl-l-methionine via metabolism to methionine. Therefore, CDP-choline could presumably recover Ptcho levels further to increasing its synthesis by impeding the PLA2 activation, which may explain the effect of CDP-choline on the ArAc content recovery of Ptetn [86]. Undoubtedly, the proposed mechanisms have helped us understand better the protective effect of CDP-Choline in cerebral ischemia but, these compounds should probably still be evaluated for evidence of their role against inflammatory processes.

Another interesting molecule that actively participates in choline donor activity is α-glycero-phosphorylcholine (α-GPC). In fact, α-GPC may be considered one of the choline-containing lipids. The role of this molecule has evaluated since the 1990s [87,88]. However, the nutritional importance of α-GPC through its combination with DHA plus triglyceride (DHA-TG) was only recently studied [89]. The results of this research have demonstrated that the α-GPC combination with DHA-TG is more neurodevelopmentally effective than DHA-TG or DHA-TG+ phosphocholine or DHA+ phosphocholine [89]. In addition, a number of studies suggest that α-GPC may represent an interesting molecule to improve some symptoms of neurodegenerative disorders which involve cognitive functions [90,91,92,93,94,95,96,97,98].

Intriguingly, α-GPC was also studied as a peripheral anti-inflammatory agent in a rodent model of small intestinal ischemia-reperfusion (IR) injury [99]. The authors concluded by affirming that their results clearly demonstrated that exogenous α-GPC administration decreased the multifactorial macro- and microcirculatory dysfunction, and lowered reactive oxygen and nitrogen species formation and ATP depletion caused by an IR insult. This evidence is in line with other findings regarding the anti-inflammatory action of α-GPC at the cerebral area [96]. Therefore, these combined data provide further indirect evidence that the anti-inflammatory role of PC may be dependent on a reaction involving the polar moieties of this molecule. It is quite clear that further investigations are required to analyze the effects of α-GPC, but it is plausible that α-GPC or its metabolites may be crucial anti-inflammatory agents if present in an inflammatory context [99].

## 6. Conclusions

The analysis of different families of lipids and phospholipids clearly suggest that these compounds actively participate in cellular life. Thus, lipids and/or their derivatives may affect every type of cells, including neurons, positively or negatively. Someone of them like sphingolipids, its receptors, and choline-containing lipids are more involved in nervous system protection. Among different proposed mechanisms for these compounds it seems that the anti-inflammatory action could be considered as the main protective machinery. Although some lipid compounds may behavior as pro-inflammatory agents (e.g., classical eicosanoids), there are many lipids that work as potent anti-inflammatory agents (e.g., phosphatidylethanolamine derivatives).

The key anti-inflammatory components of phospholipids may be their derivatives. Actually, the anti-inflammatory effects of phospholipids (e.g., lysoPC derivatives) are contentious. Thus, some of them (e.g., phosphatidylinositol) behave as an anti-inflammatory drug, suppressing CD4+ T cells, whereas others (e.g., lysophosphatidylcholine) act as pro-inflammatory molecules [100]. Conversely, their derivatives (e.g., 1-(15-hydroxyeicosapentaenoyl)-lysoPC or 1-arachidonoyl-lyso-phosphatidylcholine) act as anti-inflammatory agents through their conversion into antiinflammatory lipoxin-type derivatives in vivo [101].

On the other hand, a particular neuroprotection effect of CDP-choline and α-GPC in post-stroke models and “patients” may be explained through an anti-inflammatory mechanisms of these lipids. Actually, these compounds should be better evaluated both in cerebrovascular impairment and in other cognitive disorders (e.g., AD) where an inflammatory process, due to the presence of neurofibrillary tangles and plaques, appears to be strongly involved. 

## Figures and Tables

**Table 1:** S1P receptor subtypes and their roles in different districts.

***Immune Cells***			
**S1P Receptor Subtypes**	**Localization**	**Function(s)**	**Reference**
S1P_1_	B and T Cells NK	Monocytes circulating modulation; monocytes activation; lymphocyte differentiation	Aoki et al., 2016 [19]
S1P_2_	Mast Cells; NK?	Opposite function of S1P_1_ and S1P_3_; Inhibits early airway T cell recruitment	Aoki et al., 2016 [19] Bode and Graler, 2012 [20]
S1P_3_	B cells; Endothelial cells; dendritic cells	Chemotaxis of macrophages in vitro and in vivo	Aoki et al., 2016 [19] Bode and Graler, 2012 [20]
S1P_4_	T cells, NK?	Migration of neutrophils from blood to tissue	Aoki et al., 2016 [19]
S1P_5_	NK	Recruitment of NK	Aoki et al., 2016 [19]
***Cardiovascular System***			
**S1P Receptor Subtypes**	**Localization**	**Function(s)**	**Reference**
S1P_1_	Left and right atrium and ventricle	Possible role of cardioprotection in global ischemia-reperfusion injury; Possible role in cardiac fibrosis; heart rate	Ahmed et al., 2017 [21] Vestri et al., 2017 [22]
S1P_2_	Left and right atrium and ventricle	Possible role of cardioprotection in global ischemia-reperfusion injury; Possible role in cardiac fibrosis	Ahmed et al., 2017 [21] Vestri et al., 2017 [22]
S1P_3_	Left and right atrium and ventricle	Possible role of cardioprotection in global ischemia-reperfusion injury; Possible role in cardiac fibrosis, heart rate	Ahmed et al., 2017 [21] Vestri et al., 2017 [22]
S1P_4_	Not detected		Ahmed et al., 2017 [21]
S1P_5_	Not detected		Ahmed et al., 2017 [21]
***Respiratory System***			
**S1P Receptor Subtypes**	**Localization**	**Function(s)**	**Reference**
S1P_1_	Lung ++++	Possible role in the Airway hyper-reactivity	Kays et al., 2012 [23] Trifilieff and Fozard, 2012 [24]
S1P_2_	Lung +++	?	Kays et al., 2012 [23]
S1P_3_	Lung ++	?	Kays et al., 2012 [23]
S1P_4_	Lung +	?	Kays et al., 2012 [23]
S1P_5_	Lung +	Possible role in the progression of COPD	Kays et al., 2012 [23] Cordts et al., 2011 [25]
***Hepatic Cells***			
**S1P Receptor Subtypes**	**Localization**	**Function(s)**	**Reference**
S1P_1_	At subcellular level (nuclei and cytoplasm) +	Possible role in pathogenesis and cancer	Wang et al., 2014 [26]
S1P_2_	At subcellular level (cytoplasm)	Possible role in pathogenesis and cancer	Wang et al., 2014 [26]
S1P_3_	At subcellular level (nuclei) ++	Possible role in pathogenesis and cancer	Wang et al., 2014 [26]
S1P_4_	At subcellular level (cytoplasm)	Possible role in pathogenesis and cancer	Wang et al., 2014 [26]
S1P_5_	At subcellular level (nuclei) +++	Possible role in pathogenesis and cancer	Wang et al., 2014 [26]
***Reproductive System***			
**S1P Receptor Subtypes**	**Localization**	**Function(s)**	**Reference**
S1P_1_	Human granulosa lutein cells (hGCs)	?	Becker et al., 2011 [27]
S1P_2_	Human granulosa lutein cells (hGCs)	?	Becker et al., 2011 [27]
S1P_3_	Human granulosa lutein cells (hGCs)	Stimulatory Effects of S1P on hGCs Migration	Becker et al., 2011 [27]
S1P_4_	Not detected	?	Becker et al., 2011 [27]
S1P_5_	Human granulosa lutein cells (hGCs)	?	Becker et al., 2011 [27]

**Table 2:** S1P expression and roles in the neural cells.

Neural Cell Types	S1P Receptors Subtypes Expression	Roles
**Neurons**	S1P_1_, S1P_2_, S1P_3_, S1P_5_	Neurogenesis, Neuronal Precursors Cell Migration, Synaptic Activity, and Viability
**Microglia**	S1P_1_, S1P_2_, S1P_3_, S1P_5_	Cytokine and Growth Factor Production
**Astrocytes**	S1P_1_, S1P_2_, S1P_3_, S1P_5_	Growth Factors Production, Proliferation, Migration and Inter cellular Communication
**Oligodendrocyte Precursor Cells**	S1P_1_, S1P_3_, S1P_5_	Differentiation, Migration, Process Prolongation/Shortening, and Viability
**Oligodendrocyte**	S1P_1_, S1P_3_, S1P_5_	Inter neuronal Communication, and ??
